# Responsibility Beyond the Black Box: A Medical Student’s Reading of Giubilini’s Paper “It Is Not About AI, It’s About Humans”

**DOI:** 10.1007/s11673-026-10566-4

**Published:** 2026-05-20

**Authors:** Anna-Katharina Große

**Affiliations:** Medical Faculty, University of Augsburg, Stenglingstraße 2, 86156 Augsburg, Germany

The title of Alberto Giubilini’s article, “It is not about AI, it’s about humans. Responsibility Gaps and Medical AI” (2025) suggests that the ethical challenges of artificial intelligence (AI) in medicine are less about the technical nature of algorithms and more about our evolving understanding of human responsibility. In this commentary, I reflect on Giubilini’s arguments by situating them within a concrete clinical encounter from my medical training in cardiology.

In a rather casual conversation, my supervising cardiologist remarked that I might be among the last generations of medical students to learn auscultation properly, as this skill would increasingly be taken over by AI systems. This shift is reflected in recent studies on AI-based stethoscopes showing that algorithms can identify certain valvular heart diseases (VHD) with high diagnostic accuracy, in some settings even rivalling the performance of experienced clinicians (Zhou et al. 2025).

At first, this appeared to be merely a description of an ongoing technological evolution. But the more I reflected on it, the clearer it became that it pointed to a deeper ethical question: What happens to responsibility when we rely on “black-box” systems that may prioritize predictive performances over explainability (Kiener 2022; London 2019)? Or, put more sharply: Is understanding a necessary condition for responsibility? This question points to the core of the “responsibility gap” debate, where the loss of predictive control over learning automata is often seen as a barrier to ascribing moral agency (Matthias 2004).

I first approached Giubilini’s text guided by the sceptical intuition that opacity and a loss of control necessarily undermine responsibility. Against the backdrop of these concerns, Giubilini’s article offers a necessary conceptual shift. His aim is not to assess the technical performance of AI but to provide a framework for how responsibility remains meaningfully distributed within healthcare contexts involving AI. A central claim is that AI systems cannot be moral agents: they lack the capacity for moral reasons and thus cannot be blameworthy. This clarifies that AI does not remove responsibility from humans but forces us to redistribute it. This redistribution addresses the challenge of epistemic opacity, often described as the “black-box” nature of AI in clinical decision-making (London 2019). Rather than arguing that such opacity does not undermine responsibility, Giubilini treats this as one possible assumption and explores its implications alongside the contrasting view that opacity can, in fact, erode responsibility. To illustrate this first assumption, we can draw on London’s (2019) argument that medical decision-making has always involved elements that are not fully transparent, including human judgment shaped by experience and heuristics, and yet physicians remain responsible for their decisions.

This perspective redirected my thinking: the ethical core is not the opacity itself but how it is managed professionally. Applied to my cardiology example, this suggests that even if I cannot explain how an AI system classifies heart sounds, I may still bear responsibility for relying on its output. This justification rests on an active choice to trust a tool based on its verified clinical validity rather than a complete understanding of its code.

At this point, Giubilini’s distinction between different dimensions of responsibility becomes particularly helpful. Drawing on Shoemaker (2011), he distinguishes between attributability, answerability, and accountability.

First, regarding attributability: if the AI system were to misclassify a diastolic murmur, such as an aortic insufficiency, this technogenic error cannot be attributed to the doctor as a moral character flaw. Because the process within the black box is inaccessible, its proximal cause lies in the system’s internal functioning rather than the physician’s negligence (Matthias 2004; Santoni de Sio and Mecacci 2021).

However, the physician remains answerable to the patient and must be able to justify the decision to use the AI. This justification does not rest on understanding the algorithm itself but on the tool’s verified reliability in clinical practice. In this sense, the physician bears responsibility for what Fischer and Ravizza (1998) would call the “normative bridge” between the technical output and the clinical decision.

Extending Giubilini’s framework beyond individual clinicians, accountability shifts to the institutional level. If medical training, for example, at universities deprioritizes manual auscultation in favour of AI, as my supervisor predicted, the accountability for resulting risks lies with the system. This can be understood by applying Danaher’s concept of “tragic choice” to institutional contexts, where institutions are confronted with situations that require balancing competing moral goods (Danaher 2022). In the case of auscultation, this tragic choice concerns the question of whether to prioritize superior diagnostic accuracy through AI-supported analysis or the preservation of traditional bedside skills.

This move reveals that the “responsibility gap” is located at the systemic level, where curricula define the professional identity of the next generation. This raises a fundamental normative question: What counts as medical competence under such conditions and which forms of responsibility are future physicians expected to develop? In this sense, AI functions as a mirror: it reflects the ways in which responsibility is structured, distributed, and sometimes obscured within healthcare systems.

In retrospect, I read the article less as an attempt to resolve ethical concerns about AI and more as an invitation to reflect on the nature of human responsibility itself. My initial intuition that AI might undermine responsibility has not disappeared, but it has shifted. It is now directed less at individual clinicians and more at the systems within which responsibility is shaped, taught, and distributed.

Before closing, I would like to add one final thought. Whether or not we are currently facing a genuine responsibility gap, I believe we have a duty to prevent such gaps from emerging or widening in the future. This requires more than technical regulation; it calls for ongoing reflection on our relationship with AI and on the healthcare systems in which we operate. Even if AI systems cannot enter into relationships with us, we enter into relationships with them through the ways we rely on them and allow them to shape our healthcare systems and professional identities (Santoni de Sio and Mecacci 2021). Remaining attentive to how these relationships reshape our understanding of responsibility at a professional and systematic level may be one of the most important ethical practices for future physicians and healthcare systems (Kerasidou et al. 2022).

## Data Availability

Not applicable.
